# Clinical Efficacy of Short-Term Peripheral Nerve Stimulation in Management of Facial Pain Associated With Herpes Zoster Ophthalmicus

**DOI:** 10.3389/fnins.2020.574713

**Published:** 2020-09-23

**Authors:** Rui Han, Gangwen Guo, Yuncheng Ni, Ziyang Wang, Liuqing Yang, Jianping Zhang, Xuelian Li, Rong Hu, Dong Huang, Haocheng Zhou

**Affiliations:** ^1^Department of Pain, The Third Xiangya Hospital and Institute of Pain Medicine, Central South University, Changsha, China; ^2^Hunan Key Laboratory of Brain Homeostasis, Central South University, Changsha, China

**Keywords:** neuromodulation, peripheral nerve stimulation, facial pain, herpes zoster ophthalmicus, postherpetic neuralgia

## Abstract

**Objective:**

Peripheral nerve stimulation may be an alternative option to treat severe facial pain. We assessed the application of peripheral nerve stimulation for pain management in patients with herpes zoster ophthalmicus.

**Method:**

A retrospective analysis was conducted in patients suffering severe facial pain caused by ophthalmic herpetic lesions. We identified the change in pain severity before and after peripheral nerve stimulation for up to 12 months.

**Results:**

Eighteen patients were enrolled. Their mean age was 70.8 ± 9.5 years. Fifteen patients presented with subacute pain for 1–3 months, and three patients suffered postherpetic neuralgia. Dramatic relief from pain was achieved in 83% of patients (15 out of 18) upon initial removal of the stimulator, with pain reduction of > 50%. The long-term analgesic effect was reported at the 6- and 12-month follow-ups, with reductions in the visual analog scale of 4.8 ± 1.2 (*n* = 18) and 5.4 ± 1.4 (*n* = 11), respectively. The prevalence of postherpetic neuralgia was 7% (1 out of 15) in the subacute pain group. No obvious adverse effect was observed.

**Conclusion:**

Peripheral nerve stimulation may be an efficacious and safe approach for pain control in patients with herpes zoster ophthalmicus.

## Introduction

Herpes zoster ophthalmicus (HZO) is a viral disease associated with the first branch of the trigeminal nerve [i.e., ophthalmic nerve (V1)]. It has been estimated that about 10–20% of herpetic infections affect V1 (Liesegang, 2008). HZO prevalence has increased 3.6% per year in the last two decades in the United States (Kong et al., 2020). Patients suffer severe facial pain in the distribution of the ophthalmic division. In addition, the incidence of postherpetic neuralgia (PHN, which is defined as a persistent neuropathic pain condition) is much higher with ophthalmic involvement (Coen et al., 2006). The chronicity of the disease results in ongoing treatment in ∼20% of HZO patients (Tran et al., 2016).

Routine medical therapy for HZO comprises antiviral drugs, topical corticosteroids, and analgesics (Johnson et al., 2015). Early antiviral treatment is essential to reduce acute pain and prevent PHN development (Wood et al., 1996; Chang et al., 2019). However, conventional medications may not provide satisfactory relief from pain. Pain severity and PHN risk also increases with advancing age and immunosuppressive status, which significantly reduces the quality of life (Liesegang, 2008; Johnson and Rice, 2014; Zhou et al., 2018). Thus, it is necessary to improve pain management for HZO patients.

Recently, peripheral nerve stimulation (PNS) has increasingly been used to treat intractable headache disorders such as migraine and cluster headache through the stimulation of the occipital nerve (Saper et al., 2011; Dodick et al., 2015). Additional indications for PNS include classical trigeminal neuralgia, trigeminal neuropathic pain, complex regional pain syndrome, and fibromyalgia (Hassenbusch et al., 1996; Johnson and Burchiel, 2004; Thimineur and De Ridder, 2007; Jeon et al., 2009; Klein et al., 2016). However, the evidence for using PNS against HZO is limited. Other neuromodulation approaches, such as pulsed radiofrequency, have been widely used to treat herpetic pain (Wan et al., 2019; Ding et al., 2019). Compared with pulsed radiofrequency, PNS provides enduring pain relief for the prolonged duration of stimulation (McMahon et al., 2019). Thus, PNS may be promising treatment for HZO patients who have not responded successfully to conventional therapy.

The branches of the trigeminal nerve as well as the Gasserian ganglion are considered the targets of PNS therapy for facial pain (Kustermans et al., 2017). Specifically, by placing electrodes close to the nerves, current flow is generated between the cathode and anode to interrupt the processing of pain signals. Patients may suffer paralysis in the pain-related region instead of pain. PHN is observed in > 50% of HZO patients, mostly with involvement of the ophthalmic branch (Liesegang, 2008). The stimulation of the supraorbital and supratrochlear nerves may be an alternative pain management option for HZO patients. Here, we analyzed the effect of PNS on pain management for patients with HZO.

## Materials and Methods

### Ethical Approval of the Study Protocol

The evaluation of patient medical records was approved by the ethics board of the Third Xiangya Hospital of Central South University (Changsha, China).

### Participants

Patients’ medical records were reviewed to identify those who were diagnosed with HZO and who subsequently underwent PNS between 2018 and 2019. The diagnosis of HZO was made according to the characteristic vesicular rash and dermatomal pain associated with the distribution of V1. One brief questionnaire was given to the patients to evaluate general information, duration of preoperative pain, medication use, and postoperative pain relief.

### Implantation

**FIGURE 1:**
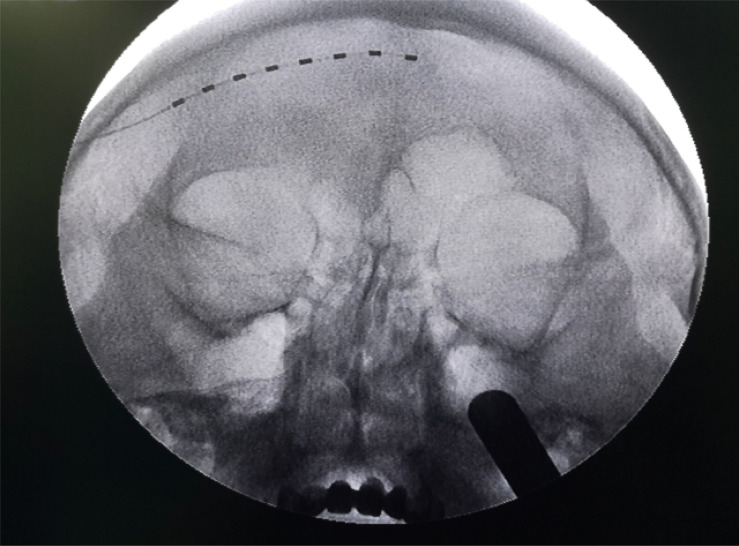
Placement of an implantable stimulation tetrode identified by fluoroscopic imaging.

A detailed description of the PNS procedure has been described by Lerman et al. (2015). Briefly, patients were placed supine with the head turned to the contralateral side of the surgical approach. Local anesthesia was induced with 1% lidocaine in a total volume of 10 mL by infiltration 2 cm lateral to the orbit at the level of the supraorbital ridge and superficial temporal fascia. A 14-G Tuohy needle was inserted 2 cm posterolateral to the junction of the frontal and zygomatic portion of the orbital rim. The Tuohy needle was advanced through the tissue and aimed at a supra-periosteal tissue plane over the eyebrow in a semilunar path, cephalad to the orbicularis oculi, terminating slightly in the cranial midline. A test stimulation lead with eight electrodes (Model 3873; Medtronic, Minneapolis, MN, United States) was inserted through the Tuohy needle. Fluoroscopic imaging was used to identify the eight-contact lead overlying the supraorbital ridge (Figure 1), and the guiding needle was removed after the lead had been placed in the desired location. The distal ends of the inserted electrodes were connected to an extension multi-lead cable (Model: 355531; Medtronic). To program the implantable electrodes, the external cable was plugged into a neurostimulator (Model 37022; Medtronic).

### Stimulation Parameter

The PNS electrodes were retained for ≤ 14 days to avoid potential infection. The duration of electrode implantation for each patient is shown in Table 1. The stimulation parameters were adjusted twice a day on the ward according to the pain assessment and tolerance of stimulation. The stimulation amplitude was set at 0.5–3.0 mV, with the painful area covered completely. The stimulation frequency was set at 40 Hz and the pulse width was 450–500 μS. The lead was programed with its own bipolar configuration. The first four tetrodes were set as the anode and the last four as the cathode. Using this stimulation strategy, we could adjust the parameter of stimulation to achieve optimal coverage of the pain region.

**TABLE 1:** General characteristics of patients.

Sub-acute pain										

Age	Sex	Area	Duration	FU	Hospitalization	Stimulation	VAS	Pre Anti-virus	Pain management	
(Years)			(Months)	(Months)	(Days)	(Days)	Pre/last FU		Pre PNS	Last FU
63	M	Rt V1	1	12	22	10	8/1	ACY	GBP, Ops, NB	Herb, acupuncture
67	F	Rt V1	1	12	17	12	8/1	ACY	GBP, Ops, NB	Herb
64	M	Lt V1	1	12	18	15	6/1	N/A	GBP, Ops, NB	GBP
71	M	Rt V1	1	12	19	10	6/2	ACY	PGB, Ops	N/A
76	M	Rt V1	3	12	24	13	8/2	ACY	PGB, Ops, NB	N/A
50	F	Rt V1	1	12	17	14	8/3	ACY	GBP, Ops, NSAIDs	None
65	M	Rt V1	1	12	17	10	8/2	ACY	GBP, Ops, NB	None
73	M	Lt V1	0.5	12	20	14	8/3	VAL	GBP, Ops, NB	None
75	F	Lt V1	1	12	21	16	6/4	ACY	GBP, Ops, NSAIDs, NB	None
57	F	Lt V1,2	1	6	15	9	6/2	ACY	PGB, Ops	PGB, Herb
62	F	Lt V1,2	1.5	6	7	14	6/2	ACY	GBP, NSAIDs	None
82	M	Lt V1	0.5	6	19	14	8/2	ACY	PGB, LP, NB	None
72	M	Lt V1,2	1	12	15	14	8/2	VAL	PGB, NSAIDs, NB	None
76	M	Rt V1	1	6	13	12	8/2	ACY	PGB, Ops, NB	PGB
80	F	Rt V1	1	6	11	10	7/1	GAN	PGB, NSAIDs, NB	None
**Postherpetic neuralgia**										
86	M	Lt V1,2	96	6	14	12	8/5	N/A	PGB, LP	None
71	F	Lt V1	72	6	6	14	8/3	N/A	GBP, NSAIDs	None
84	M	Rt V1	4	12	13	10	8/2	None	PGB, NSAIDs	N/A

**ACY, Acyclovir; GBP, Gabapentin; GAN, Ganciclovir; FU, Follow-up; Lt, Left; LP, Lidocaine Path; NSAIDs, Non-steroidal Anti-inflammatory Drugs; NB, Nerve Block; Rt, Right; Ops, Opsioids; PGB, Pregabalin; VAL, Valaciclovir.**

### Measurement and Follow-Up

The primary outcome was the change in pain intensity after PNS. This was evaluated by a visual analog scale (VAS) ranging from 0 (“pain free”) to 10 (“worst pain imaginable”). The postoperative VAS score was compared with that at baseline before implantation, and a decrease in the VAS score indicated pain relief. Patients who had pain relief ≥ 50% on the VAS after PNS were classified as “responders.” To identify the long-term analgesic effect of PNS, a telephone interview was conducted 1, 3, 6, and 12 months after discharge from hospital. “PHN” was defined as chronic neuropathic pain with a VAS score > 3 and symptom duration > 3 months, as described previously (Johnson and Rice, 2014).

### Statistical Analyses

Descriptive analysis was used to capture the general characteristics of patients. Variables are presented as the mean ± standard deviation. Paired comparisons of pain scores before and after PNS were made using the Wilcoxon signed-rank test. *P* < 0.05 was considered significant. Data were analyzed using Prism v8 (GraphPad, San Diego, CA, United States).

## Results

### Demographic Features

Eighteen patients (11 male and seven female) who received PNS therapy were identified. The mean age of patients was 70.8 ± 9.5 (range, 50–86) years. All patients presented with severe pain associated with the ophthalmic nerve after an initial herpetic skin rash, characterized by a burning, throbbing, stabbing, shocking pain. Most (15 out of 18) cases had suffered subacute facial pain for 1–3 months. Three patients developed chronic postherpetic neuralgia over 3 months. Thus, we divided the cohort into two subgroups according to symptom duration (Table 1). Fifteen of 18 participants had received antiviral treatment. One or more medications, or nerve block, was administrated before PNS. Patients were insensitive to conventional therapy, with a mean baseline VAS of 7.4 ± 0.9 (Table 2).

**TABLE 2:** Pain scores collected at each follow-up visit.

	Baseline	Discharge	1 month	3-month	6-month	12-month
VAS	7.3 ± 0.9	3.3 ± 0.8	3.3 ± 0.9	2.9 ± 1.0	2.6 ± 0.9	2.1 ± 0.9
*n*	18	18	18	18	18	11

### Follow-Up

All patients were followed up on at 1, 3, and 6 months after their hospital discharge. A final follow-up was accomplished for 11 out of 18 patients, at 12 months after PNS. The pain scores collected at each follow-up are given in Table 2.

### Therapeutic Efficacy

#### Initial Relief From Pain

**FIGURE 2:**
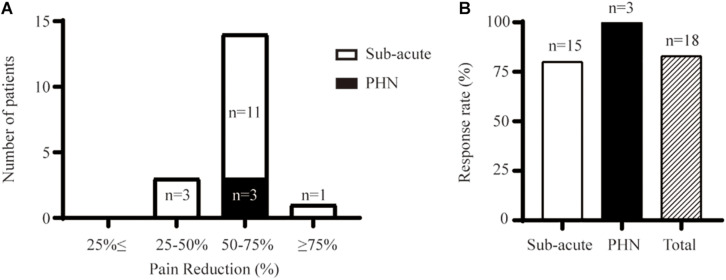
Initial relief from pain provided by PNS upon discharge. **(A)** The distribution of pain change for the subacute pain group and PHN group. **(B)** Ratio of patients having pain reduction > 50%.

**FIGURE 3:**
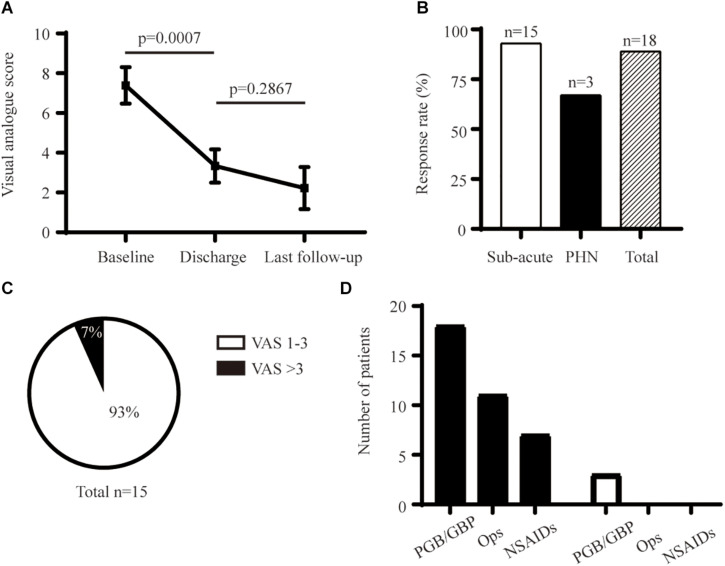
Long-term analgesic effect of PNS. **(A)** Comparison of initial and long-term pain relief. **(B)** Response rate at final follow-up. **(C)** Percentage of PHN in the subacute-pain group at a 6-month follow-up. **(D)** Detail of analgesic medication before and after PNS. GBP, Gabapentin; NSAIDs, Non-steroidal Anti-inflammatory Drugs; Ops, Opsioids; PGB, Pregabalin.

The overall response rate was 83% (15 out of 18) upon initial removal of the stimulator (Figure 2B). The response rate was 100% (*n* = 3) in the PHN group and 80% (12 out of 15) for the subacute-pain cohort. PNS provided instant and significant pain relief compared with the pain measured at the baseline (Figure 3A). Two patients with subacute pain due to HZO continued to present moderate facial pain upon discharge, with the VAS score decreasing from 8 to 5. PNS treatment provided dramatic relief from pain in all PHN patients, and the mean reduction in pain severity was 4.8 points. The initial relief from pain of PNS is shown in Figures 2, 3A.

#### Long-Term Analgesic Effect

PNS provided enduring relief from pain for 6–12 months, with an average decrease in the pain score of 5.1 (Figure 3A). The average reduction in pain was 4.8 ± 1.2 points (*n* = 18) at the 6-month follow-up and 5.4 ± 1.4 points (*n* = 11) at 12-month follow-up, respectively (Table 2). One patient who had PHN for > 8 years had recurrent moderate-to-severe facial pain 1 month after PNS. The long-term response rate was 93% (14 out of 15) and 67% (2 out of 3) for the subacute pain group and PHN group, respectively (Figure 3B). The prevalence of PHN (defined as persistent moderate or severe pain for > 3 months) was 7% in the subacute pain group (Figure 3C). Only one of 15 patients with subacute pain continued to feel mild-to-moderate pain (VAS score = 4) at the final follow-up. Use of analgesic medication was reduced after PNS (Figure 3D). Use of neither opiates nor non-steroidal anti-inflammatory drugs (NSAIDs) was reported at the final interview. Two patients continued to take pregabalin, and one was taking gabapentin with tolerable pain. Other interventions included acupuncture and treatment using herbal formulations.

#### Complications

No obvious adverse effects (e.g., infection, hemorrhage, or tetrode migration) were observed in this study.

## Discussion

Patients suffer severe and long-lasting facial pain with HZO. The high risk of PHN is associated with ophthalmic involvement (especially for older people). Conventional intervention remains insufficient to control HZO pain. Thus, improving the pain management of HZO patients and preventing PHN development are important tasks.

Early antiviral therapy can attenuate acute herpetic pain and reduce the risk of PHN (Cobo et al., 1986; Wood et al., 1996). However, many patients continue to suffer intolerable pain after conventional treatment. In the present study, 14 out of 15 patients took routine antiviral drugs in the initial phase of herpetic infection, and all of them continued to suffer severe facial pain 1–3 months later. To control pain, anticonvulsants, tricyclic antidepressants, corticosteroids, opioid analgesics, or topical lidocaine paths are recommended (Gintautas et al., 2002; Gan et al., 2013). Along with side effects (e.g., gastrointestinal and psychiatric dysfunction, addiction, and tolerance), current medications for HZO-related pain remain unsatisfactory. The blockade of the stellate ganglion may be an easy and safe option to relieve HZO-related pain, but the long-term analgesic effect is controversial (Opstelten et al., 2004).

Multimodal analgesia plays an important role in solving the undertreatment of pain. Neuromodulation therapy is a key component of a multimodal-analgesia strategy, and has been used to alleviate intractable neuropathic pain, such as “phantom limb pain” (Campbell and Long, 1976). Meanwhile, functional electrical stimulation (FES) has also been applied widely for: treatment of bladder, bowel, and sexual dysfunctions; respiration problems; restoration of hand grasping and release; and standing and stepping (Ragnarsson, 2008; Creasey and Craggs, 2012). The other clinical indications for FES include bladder prostheses, cochlear implants, retinal and visual prostheses, and vagus-nerve stimulation for the treatment of epilepsy and depression (Peckham and Creasey, 1992; Milby et al., 2008; Goetz and Palanker, 2016).

Recent innovations in PNS have indicated its potential role in the treatment of intractable facial pain (Nayak and Banik, 2018). However, few studies have used PNS specifically for the management of HZO-related pain. The effect of PNS on facial-pain control may vary based on the etiology. Johnson and Burchiel (2004) reported that patients with posttraumatic trigeminal neuropathy responded better than a cohort with trigeminal PHN. The response (pain relief ≥ 50%) was 100% in the posttraumatic neuropathy subgroup, and 50% in the PHN cohort, respectively. Likewise, we found that 2 out of 3 PHN (67%) patients with ophthalmic involvement had pain reduction > 50% at the final follow-up. Given that permanent implantation increases the risk of adverse effects (e.g., infection, electrode migration, inadequate pain relief), our data suggest that temporary PNS therapy may be an alternative option for patients with ophthalmic PHN who refuse permanent implantation of tetrodes.

Timely management of pain is crucial to prevent the chronicity of HZO. Our study focused, for the first time, on a population of patients with subacute HZO-related pain who received PNS. We demonstrated that 80% (12 out of 15) of patients with subacute HZO-related pain experienced > 50% pain relief when discharged. Only one out of 15 patients (7%) developed PHN in the subacute group 3 months after the initial skin rash. Borkar et al. (2013) found that 21% of HZO patients developed PHN eventually. The risk of PHN was 2.5-fold higher for those aged > 65 years, and only 3.6% of HZO patients aged < 45 years suffered PHN. Other risk factors of PHN associated with HZO were keratitis, conjunctivitis, and/or uveitis. Considering the advanced age of patients enrolled in our study, early PNS intervention in the subacute phase may be the key to improving the long-term outcome of HZO patients (especially for the older population).

Our study had three main limitations. First, it was retrospective. Second, it had an uncontrolled design. Third, the details of analgesic use were not recorded systematically. Randomized controlled trials are needed to confirm our findings of the superiority of PNS compared with conventional treatments for HZO-related pain.

## Conclusion

PNS was demonstrated to achieve instant and effective pain control for HZO patients. The long-term effect of PNS was enduring, with a low prevalence of PHN. For HZO patients who suffer severe pain with conventional therapy, PNS may be a promising option.

## Data Availability Statement

The original contributions generated for this study are included in the article/supplementary material, further inquiries can be directed to the corresponding author/s.

## Ethics Statement

The studies involving human participants were reviewed and approved by the ethics board of the Third Xiangya Hospital, Central South University. Written informed consent was signed before PNS therapy. The procedures and data analysis in this study were conducted in accordance with the research guideline of Third Xiangya Hospital Institutional Review Board.

## Author Contributions

HZ and DH designed the study. RuH, GG, YN, JZ, and DH carried out the procedures. XL, ZW, LY, and RoH conducted the follow-ups. XL and HZ conducted the data analyses. HZ wrote the manuscript. All authors contributed to the article and approved the submitted version.

## Conflict of Interest

The authors declare that the research was conducted in the absence of any commercial or financial relationships that could be construed as a potential conflict of interest.
